# Unemployment and Psychological Distress among Young People during the COVID-19 Pandemic: Psychological Resources and Risk Factors

**DOI:** 10.3390/ijerph17197163

**Published:** 2020-09-30

**Authors:** Netta Achdut, Tehila Refaeli

**Affiliations:** Spitzer Department of Social Work, Ben- Gurion University of the Negev, Beer-Sheva 84105, Israel; tehilarefaeli@gmail.com

**Keywords:** unemployment, psychological distress, COVID-19, psychological resources, risk factors

## Abstract

In the wake of COVID-19, unemployment and its potential deleterious consequences have attracted renewed interest. We examined (1) the association between unemployment, occurring upon the coronavirus outbreak, and psychological distress among Israeli young people (20–35-years-old); (2) the associations between various psychological resources/risk factors and psychological distress; and (3) whether these resources and risk factors were moderators in the unemployment-psychological distress link. A real-time survey based on snowball sampling was conducted during the month of April 2020 (N = 390). We employed hierarchical linear models to explore associations between unemployment, psychological resources, risk factors, and psychological distress. Unemployment was independently associated with greater psychological distress. Perceived trust, optimism, and sense of mastery decreased psychological distress, whereas financial strain and loneliness during the crisis increased this distress. The effect of unemployment on psychological distress did not depend on participants’ resource and risk factor levels. Policymakers must develop and extend health initiatives aimed at alleviating the mental health consequences of COVID-19-related unemployment and promote labor market interventions to help young job seekers integrate into employment. These measures, which are in line with the UN sustainable development goals, should be seen as an important route to promote public health.

## 1. Introduction

On 11 March 2020, the World Health Organization [1] characterized COVID-19 as a pandemic. This pandemic has affected all aspects of human life worldwide and has manifested in increasing numbers of infected people and a rising mortality rate in several countries [2]. Due to the pandemic, many governments have imposed restrictions, of varying degrees of strictness, which have impacted people’s day-to-day lives. The Israeli policy at the beginning of the crisis was considered to be quite strict: it imposed, almost all at once, comprehensive restrictions such as not leaving home for any public spaces, staying home except when absolutely necessary to go out (i.e., work, in accordance with prescribed limits; buying food or medicine; receiving medical treatment; performing other essential activities), and a prohibition against congregating [3]. At this time, all educational institutions for children ages 0–18 were shuttered (until mid-May). Restaurants and entertainment venues, shops of all kinds, malls and shopping centers, hotels, and many others businesses (excluding those defined as essential, such as grocery stores and pharmacies) were forced to close at once. Although industries considered essential maintained their activity, non-essential industries were permitted to employ only a limited number of workers. Furthermore, in April, the Israeli government announced curfews and a 100-meter limit on travel from home for nonessential activities. This reality resulted, almost immediately, in a steep and rapid increase in unemployment, from a low of 3.4% in February to a peak of 26% by the end of April [4]. In a short period of a month and a half (from mid-March to the end of April), about one million employees (942,000) were registered as new job seekers at the employment service offices throughout the country [5]. Most of these new job seekers (about 83%) were employees who had been furloughed; others had been fired [6]. During the initial phase of the crisis (March–April 2020), the rate of those who voluntarily left their jobs was very low and stood at 1.6% in March, reaching only 3.9% in April (out of new job seekers registering at the employment service bureau) [6]. It is likely that voluntary unemployment at the beginning of the pandemic was related to health risks (i.e., fears of exposing one’s self to COVID-19 at work and infecting family members) or because parents had to take care of their children following the closing of educational institutions. Young people are one of the groups most affected by the crisis, not only in Israel but also in other Organisation for Economic Co-operation and Development (OECD) countries [7,8]. In Israel, out of a million new jobseekers almost a half (445,000) were young people between the ages of 18–35 [5]. Young job seekers also have a lower chance of returning to their workplaces, as many of them had been employed in economic areas most affected by the restrictions, such as restaurants, bars, event and other entertainment-related services, sales, teaching, and education and non-professional occupations [9,10]. Indeed, during the month of May, and following a relaxation of many of the most severe restrictions, only about 18.5% of these young jobseekers returned to work [6], and many of them are now reaching the end of their unemployment benefit—both because of the overall relatively short period of payment in Israel and particularly due to the even shorter entitlement period for young people [11,12]. Self-employed workers are not entitled to unemployment compensation, and an extremely limited grant program to assist them was introduced in the pandemic’s initial phase.

Unemployment, particularly when long-lasting, is known to have severe consequences for the physical and mental health of the unemployed [13,14,15]. Furthermore, the link between unemployment and poorer health, including aggregate measures of population health, is strengthened by macroeconomic crises [16,17]. Thus, high and continuous unemployment, as we are witnessing now, is a major public health concern. This study explored the association between unemployment, occurring as a result of the coronavirus outbreak, and psychological distress (PD) among young people (20–35 years of age). We further explored factors that predict either protection (trust, mastery, optimism) or risk (financial strain, loneliness) for PD. We then examined whether the effect of unemployment on PD varies across young people with different levels of protective and risk factors.

### 1.1. Unemployment and Psychological Distress

Psychological distress is defined by the American Psychological Association (APA) dictionary of psychology [18] as “a set of painful mental and physical symptoms that are associated with normal fluctuations of mood in most people… It is thought to be what is assessed by many putative self-report measures of depression and anxiety.” Psychological distress is also defined as a “form of worry, tension, unhappiness, pessimism, and so on” ([19] p. 301) and “as symptoms of depression and anxiety” ([20] p. 1538). A similar definition was used by Arvidsdotter et al. [21], referring to PD as “a state of emotional suffering associated with stressors and demands that are difficult to cope with in daily life” (p. 687). 

The unemployment–PD (or other well-being outcomes) link is well documented in the literature [13,14,22,23]. Although many unemployment health studies suffer from the limitation of a selection scenario in which chronically ill individuals have an increased risk of becoming unemployed and, as a result, individuals with such illnesses are overrepresented among the unemployed, other studies, based on longitudinal data, have been able to capture the effect of unemployment on health. The latter type of study refers to the causality scenario, in which unemployment itself triggers illness. Although causality might run in both directions, the stronger effects are from unemployment to subjective well-being [24].

Metanalytical evidence provides the clear conclusion that both selection and causality effects have a role in the mental health of unemployed individuals: unemployed people show more distress than do those who are employed, as measured by various psychological well-being indicators including mixed symptoms of distress, depression, anxiety, psychosomatic symptoms, subjective well-being, self-esteem, and life satisfaction [25,26,27]. Based on the European Social Survey (ESS), Van der Meer [28] demonstrated the negative association between unemployment and subjective well-being, which consisted of measures such as stress, fulfillment, life satisfaction, and personal happiness. Daly and Delaney [29] examined the association between the life-time duration of unemployment over a 34-year period and psychological distress at age 50 years in a sample of British adults. Their findings supported the idea of psychological scarring from unemployment affecting adult well-being. A review by Reneflot and Evensen [23] on the PD consequences of unemployment in young adults indicated that unemployment increased the risk of PD, even after initial mental health status and confounding factors were accounted for. The study of Taht and colleagues [30] showed that unemployment impaired young Europeans’ cognitive (life satisfaction) and affective (negative affect) well-being, and Thern, de Munter, Hemmingsson, and Rasmussen [31] demonstrated the long-term effects of youth unemployment on their mental health. Finally, the long-term unemployed carry a markedly higher burden of mental illness compared to employed individuals and those who are unemployed for only a short time, and the burden of disease increases with the duration of unemployment [15,17,32].

### 1.2. Theoretical Framework

Unemployment is a major source of stress, especially when the economy retracts and there are few job options. In the stress literature, unemployment is considered to be one of the main discrete and objective life events that requires some social or psychological adjustment on the part of the individual [33]. A common explanation for the relationship between unemployment and mental health, which was adopted in the current study, is the stress process model [34,35]. This theoretical model refers to the effects of stress on PD in general and suggests that being unemployed increases the probability of experiencing stress-inducing factors such as a lack of resources, limited opportunities, and low self-regard, and limits access to privileges and security [35]. That is, unemployment likely brings in its wake a range of losses, including those of social ties and economic stability, and these losses may be stressful in and of themselves [34]. Hence, unemployment increases the risk of impaired mental health. 

The literature indicates that employment serves both manifest and latent functions. The manifest or intended function is earning a living, whereas the latent or unintended functions include identity definition and connection to important social institutions and associations [36]. Accordingly, there are psychological costs of unemployment, including one’s potential loss of meaning in life, impairment of personal identity, and loss of the self-esteem that one typically draws from one’s job [37]. Unemployment is therefore stressful because it involves the loss of all of these types of benefits. However, the magnitude of unemployment on one’s PD might also depend on contextual stressors occurring at macro levels of the social reality in which the individual is embedded [33]. One well-documented chronic macro level stressor comprises recession, economic crisis, and high unemployment [17,38]—the precise scenario that occurred in the wake of the COVID-19 pandemic. 

However, various social and personal resources might act as protective factors buffering the effects of the stressor (unemployment) on distress symptoms (PD) [39]. 

The conservation of resources theory (COR) [40] offers a theoretical framework to understand how personal resources moderate the unfavorable effects of unemployment on PD. Hobfoll [40] explains that individuals will experience distress when they feel threatened with a loss of something they value (a resource) or when they actually experience a loss. The anticipated or observed loss of resources, such as job loss, could be perceived as a reduction in the ability to manage challenges, leading to a reduction in one’s well-being. The COR theory suggests four categories of resources: objects (e.g., shelter, food, transportation), conditions (e.g., secure employment), psychological characteristics (e.g., optimism, sense of mastery), and energy (e.g., time, money, knowledge). In this context, having a job is considered a conditional resource that helps individuals gain other types of resources such as objects and energies (i.e., food, shelter, money) and personal resources (e.g., mastery). When “conditions” and “energy” are lacking, individuals are unable to deal with environmental threats, and experience impairments in their well-being [40,41]. Furthermore, individuals who possess few resources are more vulnerable to the loss of resources, while those with greater resources are able to gain additional resources [41]. Thus, those with low levels of resources—of different kinds—are assumed to experience greater distress in the wake of unemployment compared to individuals with greater resources. 

### 1.3. Psychological Resources, Risk Factors, and PD

In this study, we used Hobfoll’s COR theory [40] and Pearlin’s stress process model [35] to elucidate the factors that predict either protection (i.e., sense of mastery, optimism, perceived trust, and trust in institutions) or risk (i.e., financial strain and loneliness) for PD. Mastery was defined by Pearlin and Schooler [42] as the perception that events are under one’s own personal control, rather than under the control of external forces. Individuals with a high sense of mastery believe in their power to influence the environment and bring about desired outcomes. Mastery is associated with cognitive coping strategies, which consist of mental efforts to alter one’s perceptions of stressful demands so that they seem less threatening. These strategies can reduce emotional reactions to demands [42]. Cognitive strategies are of great value in situations of macro external events that are beyond individuals’ control, such as unemployment occurring in the wake of this pandemic. To support this notion, previous studies demonstrated the modifying role of mastery in the unemployment (and financial strain)–PD link [43,44]. 

Optimism is defined as a personality construct characterized by a generalized tendency to expect positive outcomes, or the belief that ‘‘good as opposed to bad things will in general occur in one’s life’’ ([45] p. 26). One of the most important implications of this positive outlook is that optimists cope better in times of stress. When problem-focused coping is of limited use, such as in the pandemic’s initial phase in Israel, optimists will turn to more adaptive emotion-focused strategies (also referred to as cognitive coping strategies), such as a positive reinterpretation of the situation [46]. Accordingly, in their meta-analysis, Solberg Nes and Segerstrom [47] concluded that optimists switch between problem– and emotion-focused coping, depending on the stressor’s controllability. That is, optimists may be more flexible than pessimists in their choice of coping strategies. This flexibility, we assumed, would be of great importance at a time of an uncontrollable stressor, such as pandemic-caused unemployment. In addition, studies have demonstrated that optimistic individuals maintain higher levels of well-being in times of unemployment [48]. Although COR theory [40] also refers to additional psychological resources (e.g., self-esteem), we chose the measures that we assessed as core resources in the context of the pandemic. We assumed that, in times of such major and unexpected external event accompanied with increasing uncertainty (macro stressor) associated with individual stressful life events (unemployment as micro stressor), one’s perception on one’s ability to control his life along with tendency to expect positive outcomes is of great value to one’s wellbeing.

Finally, trust is a central construct of social capital, which is conceptualized as and operationalized by structural and cognitive components. Whereas the structural components involve the extent and intensity of social connections and participation that can be objectively verified, the cognitive components include, among others, the subjective perception of trust [49]. Rotenberg [50] suggests that trust is the belief that others will fulfill their promises, and the emotional experiences accompanying those beliefs or attributions (feelings of confidence in others). These beliefs might moderate the stress associated with unemployment. In line with this assumption, trust has been previously studied as a moderator in the unemployment-well-being link [51], as a mediator in the SES–PD link [52], and as a moderator in the poverty-loneliness link [53]. Given the harsh restrictions on social gatherings and activities (the structural component of social capital) and the lockdown imposed during the month of April, we thought that the cognitive component would be more appropriate than the structural one for measuring social capital.

Turning to risk factors, we explored the effect of financial strain and subjective loneliness as risk factors for PD. Pearlin [54] suggests that the strain of struggling with inadequate financial resources may foster a sense of insecurity, uncertainty, and hopelessness about the future, thus causing PD [55]. Financial strain can also be conceptualized in the context of COR [40] as a loss of energy resource. This loss, along with the limited ability to gain new financial resources in the wake of the pandemic, can in turn increase PD. Previous studies have documented the association between financial strain, particularly when long-lasting, and PD [56,57,58].

In addition, loneliness—defined as an unpleasant subjective feeling due to a perceived lack of connectedness with others—could be a result of a reduction in the quality or quantity of an individual’s social connections [59]. It could also be derived from a gap between an individual’s actual and expected number and quality of social interactions and relations [60,61]. Loneliness has been found to be a major risk factor for the mental health of people of all ages [62,63,64] and its association with PD is well documented [65,66]. This link may reflect the gap between one’s desire for vs. actual social interactions, potentially leading to an increase in PD. In the wake of the pandemic and the severe limitation on people’s social activities we would expect a greater gap between people’s desire and their actual social interactions. Moreover, Jahoda [36] and Pearlin [35] suggest that the losses associated with unemployment can lead to social isolation. Indeed, unemployment, accompanied by the loss of social ties and regularity of daily social interactions that provide a sense of belonging and support, have been found to be related to loneliness in previous studies [62].

### 1.4. The Current Study

This study examines (1) the association between unemployment, which occurred as a result of the coronavirus outbreak, and PD among young people between the ages of 20–35 in Israel; (2) the associations between various psychological resources (trust, optimism, sense of mastery) and risk factors (financial strain and subjective loneliness) with PD; and (3) whether these resources and risk factors were moderators in the unemployment-PD link. 

We hypothesized that, first, unemployment would increase PD, and this effect would decrease once we controlled for the psychological resources of trust, optimism, and sense of mastery, and for the risk factors of financial strain and loneliness. Second, greater psychological resources would be associated with decreased PD. Third, financial strain and loneliness would be associated with increased PD. Fourth, the effect of unemployment would be decreased by a greater sense of mastery, optimism, and trust. Fifth, the effect of unemployment would be greater for those reporting financial strain following the COVID-19 outbreak and for those reporting an increase in their subjective feeling of loneliness.

## 2. Method

### 2.1. Sample

The study’s final sample comprised 389 young people between the ages of 20–35 (M = 26.89, SD = 4.0). This final number was arrived at after we excluded 9.9% (n = 45) of the 454 participants who completed the questionnaire, as we were unable to identify their employment status either before or during the crisis, and 4.4% (n = 20) who reported that they were not employed at all in the month preceding the coronavirus outbreak. Most of the participants (95.1%) were born in Israel (5% were immigrants, most of them from the Former Soviet Union), and almost all of them (95.0%) were Jewish. A quarter (25.6%) were married, and 61.2% had post-secondary education. About half of the participants (45%) lived in the center of Israel, a third (35.4%) in the south, a tenth (11.3%) in the Jerusalem area, and 8% in the north. None of the participants were positive for COVID-19 in the period prior to the study or at the time of the survey.

We conducted post hoc power analyses using Gpower 3 software (Heinrich Heine University Düsseldorf, North Rhine-Westphalia, Germany) to estimate the observed power based on the following parameters [67]: α = 0.05, sample size = 389, multivariate linear regression including 18 predictors, and an effect size of 2% explained variance. The analysis indicated an 80.24% chance of detecting employment status differences in PD. We selected a conservative effect size of only 2% explained variance to allow the sensitivity of our model in the detection of small differences in PD by employment status and to cover all expected effects (small, moderate, strong). Additionally, the ratio between number of independent variables and number of cases in this study as yielded from our multivariate analysis is considered to be good (1:21–22) [68].

### 2.2. Process

Data were collected during the month of April and the first week of May 2020, a time during which harsh restrictions had been imposed in Israel. The sample was drawn using an online snowball sampling method. The researchers shared a link to the questionnaire via WhatsApp (Facebook, Inc; Mountain View, California, United States) and Facebook (Facebook, Inc; Mountain View, California, United States), including weekly postings of this link on relevant Facebook groups of young adults. We asked participants to share the link with friends and family via any social media means they preferred. Due to young people’s frequent use of social media, using the snowball sampling method was more direct and purposeful than had we used any other type of non-probability sampling method. It is worth noting that the use of online snowball sampling does not allow for an assessment of the response rate, as there is no way to determine exactly how many young people received the link and did not respond to it.

### 2.3. Measurement

#### 2.3.1. Dependent Variable 

Psychological distress (PD) was assessed using seven items referring to the COVID-19 period. These items were taken from the Israeli Social Survey (ISS) [69], and adapted for the current study. That is, the period of the five first items was changed from “the last 12 months” to “the last two months” in order to capture PD in the wake of COVID-19. The questions were (1) have you felt pressured, (2) have you felt depressed, (3) have you felt that worries prevented you from sleeping, (4) have you felt full of energy, and (5) have you felt that you are able to deal with your problems. Answers were rated on a four-point scale ranging from 1 (always) to 4 (never). Two items opened with the statement, “In the past two weeks, did it happen that,” and responses were: (6) you did not find interest in your activities, or did not feel pleasure with your activities, and (7) you were in a bad mood, felt depressed, or hopeless. Answers were rated on a scale of 1 (did not happen at all) to 4 (happened every day, or almost every day). After reversing the relevant items, a summed score for these seven items was calculated. The final answers ranged from seven to 28 points, with a higher score reflecting higher PD. Cronbach’s α indicated a high internal reliability (0.79).

#### 2.3.2. Independent Variables

*Unemployment.* Participants were divided into two groups. Employed participants (=0) were defined as those who worked before the crisis and continued to work up to the time of the survey, either as an employee or as someone who was self-employed (even if they experienced a reduction in their working hours or a reduction in the volume of business activity). Unemployed participants (=1) were defined as those who worked before the crisis, but were fired, furloughed, or had to close their businesses. It should be noted that none of the participants left their jobs voluntarily. As mentioned, 20 (4.4%) young people who were not engaged in paid work (unemployed or non-participants in the labor market) before the crisis were excluded from the final sample.

*Perceived income adequacy (PIA)* was assessed via two questions: (1) “Do you usually, in routine times, manage to cover all your monthly expenses for food, electricity, telephone, etc.?” Answers were provided on a four-point Likert scale (yes, without any difficulty = 1; yes, but with some difficulty = 2; no, not so well = 3; no, not at all = 4). Based on this item, we created a dummy variable where participants who reported that they were unable to meet their expenses—*no*, *not so well* and *no*, *not at all*—received the value (1). Similar items have been used in previous studies to evaluate one’s subjective social position [70,71]; (2) *PIA during the Covid-19 pandemic* was assessed by the question, “Has there been an adverse change in your ability to cover your household monthly expenses since the coronavirus outbreak?” (1 = yes).

*Perceived trust* was measured using a single item: “In general, can you trust most people, or do you have to be wary of them?" (1 = yes). *Trust in institutions* was measured using three corresponding items in which participants had to indicate how much trust they had in the government, justice system, and health system. Answers were provided on a four-point Likert scale ranging from 1 (yes, to a great extent) to 4 (not at all). After reversing the three items, an index was computed as the sum of the items and ranged from three to 12, with a higher score reflecting higher trust. Cronbach’s α indicated a moderate internal reliability (0.61). These items were drawn from the ISS [69].

*Sense of mastery* was derived from the measure developed by Pearlin and Schooler [42], which comprises seven statements regarding participants’ feeling of being able to control things in their lives, for example, “I have little control over the things that happen to me.” Participants were asked to indicate, on a scale ranging from 1 (strongly agree) to 5 (strongly disagree), the extent to which each statement described them. The index is calculated as the sum of the items. This scale was translated into Hebrew and validated by Hobfoll and Walfish [72]. Cronbach’s alpha was 0.88 for the original questionnaire [72] and 0.78 in the present study.

*Optimism* was measured by the Life Orientation Test (LOT-R) [73]. This scale includes 10 items with four filler items. Respondents were asked to indicate their level of agreement with each item, on a scale of 1 (strongly agree) to 4 (strongly disagree). The scale was translated into Hebrew and validated by Drori and Florian [74]. Cronbach’s alpha was 0.74 for the original questionnaire [74] and 0.83 in the present study.

*Loneliness* was measured by a standard single item: “How often have you felt lonely in the last week?” A single-item measure has proved suitable for capturing the subjective feeling of loneliness [60,75]. Answers were provided on a four-point Likert scale (1 = often; 2 = sometimes; 3 = seldom; 4 = never). We merged the categories of feeling lonely never and infrequently into one category that we designated “infrequently-never”, thereby yielding three categories of loneliness: infrequently or never felt lonely (=0); sometimes felt lonely (=1); and felt lonely often (=2). Change in the subjective feeling of loneliness were measured by, “Since the outbreak of the coronavirus crisis, have you felt lonelier than before (during routine times)?” Answers were provided on a four-point Likert scale (no, my feeling of loneliness before the crisis and today is similar = 1; yes, slightly lonelier than before = 2; yes, lonelier than before= 3; yes, much lonelier than before = 4). Based on this item, we created a dummy variable where participants who reported that they were lonelier, to any extent, than before, received the value 1.

*Control variables* (demographics) included age, gender, marital status and parenthood, education, parental education (as an indication of family background and resources), currently studying, and suffering from a health problem or a limitation that lasted at least six months.

### 2.4. Data Analysis

First, we conducted a bivariate analysis to assess the associations between the dependent variable (PD) and the study variables. ANOVA and t-tests were computed in order to assess differences in the means of PD by categorical variables. Pearson correlations were employed with respect to continuous explanatory variables. Second, a five-step hierarchical linear regression model estimated PD. In the first step, we included sociodemographic characteristics, physical health status, and PIA in routine times (Model 1). To study the distinct contribution of unemployment to PD, in the second step we added employment status (Model 2). To learn the distinct contribution of financial strain in the wake of the coronavirus outbreak to PD, in the third step we added PIA during the crisis (Model 3). In the fourth step, we added the psychological resources of perceived trust, trust in institutions, optimism and sense of mastery (Model 4). In the fifth step, we incorporated the two measures of subjective loneliness (Model 5). Finally, to examine whether the magnitude of the effect of unemployment varied among participants with different levels of psychological resources and risk factors, we added to the full regression model a sixth step with two-way interaction terms between employment status and each resource (trust, optimism, sense of mastery) and risk factor (financial strain and loneliness). Due to multicollinearity between these interaction terms and small cells constraints, we could not include all the interaction terms in a single model. Thus, we estimated our model with one interaction term at a time. Data were analyzed using IBM SPSS ver. 25 Statistics for Windows (IBM Corporation, Armonk, NY, USA). 

### 2.5. Ethical Considerations

The study followed the ethical guidelines of the authors’ university and was approved by the Ethics Committee at Ben-Gurion University of the Negev. The participants electronically provided their informed consent and were assured that their identities would remain confidential. The questionnaire itself did not include any personally identifying details. 

## 3. Results

Table 1 summarizes the descriptive statistics of the study variables in the overall sample and by employment status. As detailed in the Measurement section, being unemployed referred to those who had been working prior to the coronavirus outbreak (either as salaried workers or as self-employed individuals) and who had been furloughed or fired, as well as self-employed people whose businesses had closed. In almost all measures, there were no statistically significant differences between the two groups. This finding indicates that the newly unemployed young people did not differ from the young people who continued to work during the pandemic with respect to any of the demographic characteristics, PIA in routine times, physical health status, perceived trust, trust in institutions, sense of mastery, optimism, or subjective loneliness. An exception was marital status; namely, married young people were less likely to have become unemployed in the wake of the pandemic, perhaps as a result of employers’ tendency to keep workers who had greater family responsibilities. However, as expected, substantial differences between the groups were found in the two measures indicating a change in their situation during the crisis: Unemployed participants were more likely to report financial strain in the wake of the crisis, as measured by emerging difficulties in covering their household expenses, and they were also more likely to report an increase in their feelings of loneliness.

Table 2 presents the mean value (SD) of PD by the study variables. The mean value of PD in the overall sample stood at 15.57 (3.72), indicating relatively moderate distress. PD means values differed in almost all of the independent variables and indices, with the exceptions of age, education level, and being a student. Unemployed participants reported greater distress than those who held onto their jobs and business during the crisis (16.61 (3.85) and 15.08 (3.57), respectively), and those who reported emerging financial strain in the wake of the crisis had greater distress than did those who were able to meet their expenses (16.89 (3.74) and 14.75 (3.48), respectively). Psychological distress was also higher for participants with low perceived trust (16.75 (3.88) and 14.83 (3.41), respectively), and a great deal higher for those who reported greater subjective loneliness. For example, the mean value of participants who “never-infrequently” felt lonely was 13.30 (2.83), compared to a much higher mean among those who “often” felt lonely, 19.25 (3.07). Furthermore, an increase in the feeling of loneliness in the wake of the coronavirus outbreak was also related to greater PD (16.68 (3.65) compared to 14.24 (3.45) among those who had felt equally lonely before the crisis). As expected, a negative correlation was found between trust in institution, although weak (r = −0.18), optimism (r = −0.52), and sense of mastery (r = −0.45) to PD. 

Table 3 shows the five-step hierarchical linear regression analysis. The dependent variable in all models was PD. Coefficients (standard errors in parentheses) and standard coefficients of the independent variables are presented. According to the first step, men, married participants, and those who had two parents with an academic education had lower PD. Suffering from a health problem or a limitation and having difficulties in covering monthly expenses in routine times were positively correlated with PD. Participants’ sociodemographic characteristics accounted for 9.8% of the explained variance in PD. The second step indicated that unemployment was positively correlated with PD. This step, including employment status only, made a distinct contribution of 2.6% to the total explained variance. The third step, as expected, showed that emerging difficulties in covering monthly expenses in the wake of the crisis was positively correlated with PD, and explained 2.8% of the variance in PD. The fourth step indicated that perceived trust, optimism and sense of mastery were all negatively associated with PD. That is, having greater psychological resources was associated with decreased PD. These resources made a substantial distinct contribution to the total explained variance of the model (27.4%). To the final step, we added the loneliness measures, which indicated that both subjective loneliness and a feeling of greater loneliness during the crisis, compared to these feelings in routine times, were positively associated with PD. Loneliness, thus, was found to be a severe risk factor for PD, accounting for 12.3% of the total explained variance. It is also important to note that the coefficient of unemployment, although slightly reduced, remained significant in the final model. That is, all else being equal, unemployment negatively associated with PD. The entire set of the independent variables accounted for 54.8% of the variance in PD, F (18,364) = 24.53, *p* < 0.001. All five models made a distinct and significant contribution to the explained variance. The final regression model indicated that gender, PIA in routine times, unemployment, perceived trust, mastery, optimism, and the loneliness variables were significant predictors of PD. We found no evidence of moderation: the effect of unemployment on PD appeared to be uniform across participants, regardless of their different levels of psychological resources as well as risk factors (not shown). Estimations derived from models including non-significant interaction effects are available upon request.

## 4. Discussion 

The COVID-19 pandemic is an ongoing event associated with growing uncertainty, major economic crises worldwide, and continual effects on people’s daily and social lives. Thus, it has national and global repercussions for both the physical health and for the mental health and well-being of many [76,77,78]. Although the pandemic constitutes a low direct threat to the physical health of young people, it has direct and wide effects on young people’s lives and their well-being through, among other things, its impact on their labor market activity. In Israel, as in other Western countries, young people have been one of the groups most affected by the current economic crisis, as many of them experienced removal, either temporary or permanent, from their jobs [5,7,8,79]. Moreover, current indications show that most of them have not yet returned to work [10]. 

In this study, we explored the association between unemployment, occurring during the first wave of the pandemic, with PD among young people in Israel. We further used the COR theory [40] to identify psychological factors that can protect young people from PD, as well as risk factors that might increase it. We then explored whether these various personal resources and risk factors were moderators in the unemployment-PD association. 

Our findings strongly support the association between unemployment and PD, and they align with the knowledge that has accumulated over the years in regard to the general population [25,27,38] and to young people [23,30]. This association remains even after controlling for demographics, financial strain, emerging financial strain in the wake of the pandemic, and the various psychological resources included here. In other words, employment not only increases people’s subjective well-being by enabling them to have the income to buy goods and services, but also by satisfying greater and deeper human needs. As previously mentioned, employment contributes to personal identity, meaning in life, and connection with social institutions, and also serves as a source of self-esteem [36,37]. These factors might explain why the detrimental effects of unemployment on subjective well-being usually holds, as in the case of this study, even when income and financial strain are controlled for [80,81].

Moreover, our findings indicated that perceived trust, optimism and sense of mastery were negatively associated with PD. These findings are in line with the COR theory, which emphasizes that people who possess more robust personal resources during periods of stress are better able to cope with stressors than those who have fewer resources [41], and that these resources serve to protect their subjective well-being [82]. These resources, along with perceived trust, which is a central cognitive component of social capital [49], have proven to be important protective factors against PD, even in times of a national and global crisis.

As expected, subjective loneliness—including an exacerbation of the subjective feeling of loneliness during the pandemic—was associated with greater PD. This finding confirms previous evidence suggesting the negative effect of loneliness on the mental health of young people [83,84,85]. The worldwide lockdown and the restrictions imposed on social interactions and gatherings in the wake of the pandemic have increased and strengthened the feeling of loneliness, particularly among young adults [86], causing damage to their mental health [2,87]. Here, we found that, among the unemployed, reports of exacerbated loneliness as a result of the pandemic were higher than among those who continued to work, putting them at higher risk of impaired mental health. This finding was probably due to other losses associated with unemployment, such as the loss of sense of belonging, work-related social interactions, and the support these interactions provide. Finally, emerging financial strain during the coronavirus outbreak was more prevalent, as expected, among the unemployed and was associated with greater PD. This finding aligns with the accumulated knowledge on the financial strain-PD link [56,57,58] and also supports Pearlin’s stress process model [54], which emphasizes that inadequate financial resources substantially affect one’s PD.

Although the findings here pointed to the positive contribution of psychological resources to PD on the one hand, and the importance of the considered risk factors on the other hand, none of the psychological resources nor risk factors appeared as moderators in the unemployment-PD association. That is, the effect of unemployment on PD was neither lessened by greater psychological resources nor strengthened by emerging financial strain or greater loneliness. This line of findings runs counter to Wheaton’s theoretical model [39], which suggests that different personal and environmental resources, including financial resources, moderate the stress–distress link. They also provide only partial support for the COR theory argument. In line with COR, we found that young people with greater conditional resources (employment), energy resources (money as measured by PIA), and psychological resources (trust, optimism, mastery) were better off in terms of PD. However, our findings do not confirm the idea that individuals who possess fewer psychological resources (trust, optimism, mastery) and fewer energy resources (money) are more vulnerable in terms of their well-being to the loss of other resources (i.e., the loss of employment). That is, we found no evidence that an initial lack of resources, or alternatively greater resources to begin with, makes individuals more or less vulnerable to the negative effect of the loss of other resources they once possessed [41]. 

Our findings also contradict previous studies that indicated the moderating role played by sense of mastery, optimism, as well as less financial strain, in the unemployment–PD association [88,89]. The underlying rationale for the current findings might be that experiencing unemployment in the context of a macro event such as COVID-19, which is associated with additional contextual stressors such as a severe economic crisis and uncertainty, undermines the possible moderating role of psychological resources. In other words, personal resources, which may ordinarily serve to moderate the negative effect of unemployment on PD, are not sufficient for dealing with the stress associated with what is expected to be long-term unemployment. It is also possible that in the wake of the pandemic and its associated labor market conditions, young unemployed people could not gain resources (i.e., find new jobs) after investing existing resources (i.e., job search efforts) to gain valued resources. This inability to gain resources given one’s investment in doing so, according to Hobfoll [40], is also associated with the experience of distress. In addition, the findings of Wade-Bohleber and colleagues [90], regarding adolescents and young adults, indicated that whereas the employed participants were able to use personal resources to alleviate stress, the unemployed participants needed external support to deal with stress. It may therefore be that for young people who experienced unemployment during this crisis, only external resources (and not internal psychological resources) will be able to mitigate the unemployment-PD association. 

The current study was exploratory, providing initial insights on the effect of unemployment on PD among one of the groups most affected by the current economic crisis. Another important aspect of this study was that it overcame a limitation that typifies many unemployment health studies; because we knew the order in which events occurred, we could also capture the effect of unemployment on PD. Given that the circumstances of unemployment were related to the major economic crisis, we had no reason to assume that being in poorer mental health might have been the cause for unemployment, but rather its effect (see discussion in [17]). 

Along with its merits, this study had several limitations. First, it was based on a non-representative sample and, as mentioned, using online snowball sampling does not allow for an assessment of the response rate. Second, the study included young people from Israel’s majority group, and did not represent minority groups, such as the Arab minority. Third, it might be that other factors not included in our model could be considered as moderators in the unemployment–PD association, such as the availability of family and community support. Finally, the data were collected during the very beginning of the pandemic. Although this aspect enabled us to capture the short-term effects of unemployment on PD, the long-term effect has yet to be studied. The association between unemployment when unemployment benefits and/or other public support are no longer available must also be investigated.

## 5. Conclusions 

The COVID-19 pandemic and the accompanying economic crisis pose a significant threat to the mental health of young people. Current economic forecasts suggest that market recovery in Israel and in other countries will take a long time [9,91]. Thus, many of the newly unemployed can expect to suffer from prolonged unemployment. According to Pearlin [35], chronic stressors such as long-term unemployment have the most negative consequences for people’s PD. Similarly, prolonged unemployment, associated with increased financial strain and a further decline in one’s self-esteem, is known to have the most severe consequences for the mental health of individuals and families [15,32]. This effect is further aggravated in times of economic crisis [32]. Moreover, we can assume that other contextual stressors related to the coronavirus outbreak, such as the fear of being infected, worries about older family members, and the restrictions imposed on our social lives, might also strengthen the unemployment–PD link. Given all of the above, the current unemployment crisis presents a great public health concern, and urgent action is needed.

## 6. Recommendations

In accordance with the sustainable development goals formulated by the UN [92] to promote mental health through prevention and treatment, governmental responses should be targeted to the emotional effects of this pandemic. This goal should be achieved by developing and extending adequate public and subsidized services, such as free online counseling, online support groups, and call centers that will provide mental health assistance and will direct applicants to available community services when needed. One main focus of such interventions, as we indicated earlier, should be to alleviate loneliness. These interventions should be directed towards young people, and particularly those who have lost their jobs. Whereas the UN goals mainly refer to physical health, in general and specifically following the pandemic, we believe that the issue of mental health promotion demands an equal response in the local and global spheres.

Diminishing poverty, generally and in the wake of the pandemic, is the first goal of the next 10 years as defined by the UN [92,93]. To reduce the consequences of financial strain and deprivation additional public efforts are essential in ensuring financial aid for the unemployed, through existing social security programs (unemployment benefits and income support schemes) and measures targeted to self-employed people whose businesses have been harmed. Such measures, although to varying extents, have already been applied in many countries, such as in Israel [12,94], but given how long this economic crisis is expected to last, additional support is needed. Indeed, previous empirical evidence has indicated that public support, mainly unemployment benefits, moderate the unemployment–PD association [15,17]. 

Another of the sustainable development goals formulated by the UN [92,93], and threatened by the current crisis, is the promotion of employment and decent work for all, with young people being a target group. Here, governments are called on to protect jobs, vulnerable workers, and small and medium enterprises [94]. Accordingly, we suggest that active labor market interventions can assist young people who may not be able to return to their former line of work but are able to integrate into the job market. Fast-tracking of vocational training and skills acquisition in areas which held steady or even grew stronger during the crisis can be an effective route. Advanced employment service placements that will increase both the rate of return and job matching are necessary as well. The vicious cycle of unemployment and poor health can be broken only by the combined effects of available healthcare, special health-promoting measures among the unemployed, and public interventions aimed at relieving economic distress [17]. All three are needed now to help young adults manage this crisis both financially and mentally. 

Finally, we would suggest developing interventions to strengthen young people’s psychological resources, trust, sense of mastery, and optimism, as all were found to be important for decreasing PD. Increased perceived trust can be achieved by providing individuals with opportunities for being listened to and acknowledging their thoughts and feelings [95]. As trust is based on the feeling that people can rely on others’ help in times of need, it would seem that support provided during this crisis by social and mental health services could contribute to improved trust [96]. Sense of mastery can be enhanced by interventions that will help young people recognize their current control of their daily life as well as their ability to plan their daily life schedule [97]. These interventions can help participants see their life positively, as they will be able to identify desirable events that have already occurred in their lives, or be more active in creating positive events and avoid negative ones, which can then decrease PD [98]. Another means to increase mastery is using the positive psychology approach which highlights previous successes in life [99]. Finally, the meta-analysis of Malouff and Schutte [100] indicated that psychological intervention can improve optimism, with the most promising tool being the best possible self method, which includes focusing on imagining a positive future in which patients achieve all their personal goals.

## Figures and Tables

**Table 1:** Descriptive statistics of the study variables by employment status (N = 389).

Employment Status	Overall	Employed (N = 265)	Unemployed (N = 124)	t/ Chi-Square
	100%	68.1%	31.9%	
	%/ Mean (SD)	%/ Mean (SD)	%/ Mean (SD)	
**Age**	26.89 (4.0)	27.09 (4.16)	26.46(3.60)	t = 1.52
20–25	38.0	36.2	42.0	χ^2^ = 5.40
26–30	40.4	38.9	43.5	
31–35	21.6	24.9	14.5	
**Gender**				χ^2^ = 0.93
Male	19.8	21.1	16.9	
Female	80.2	78.9	83.1	
**Marital status**				χ^2^ = 4.38 *
Married	22.6	25.7	16.1	
Other	77.4	74.3	83.9	
**Have kids**				χ^2^ = 3.05
Yes	18.8	21.1	13.7	
No	81.2	78.9	86.3	
**Education**				χ^2^ =0.74
Secondary education	38.8	37.4	41.9	
Post-secondary education	61.2	62.6	58.1	
**Parents’ education**				χ^2^ = 0.09
Both academic	45.5	46.0	44.4	
Other	54.5	54.0	55.6	
**Currently studying**				χ^2^ = 1.77
Yes	49.1	46.8	54.0	
No	50.9	53.2	46.0	
**Health problems/ limitations**				χ^2^ = 0.02
Yes	16.7	16.6	16.9	
No	83.3	83.4	83.1	
**Perceived income adequacy in routine times**				χ^2^ = 0.62
Difficulties in covering monthly expenses	11.8	10.9	13.7	
No difficulties in covering monthly expenses	88.2	89.1	86.3	
**Perceived income adequacy during COVID-19**				χ^2^ = 63.14 **
Emerging difficulties in covering monthly expenses	38.3	24.9	66.9	
No difficulties in covering monthly expenses	61.7	75.1	33.1	
**Perceived trust**				χ^2^ = 1.08
Yes	62.8	64.5	59.0	
No	37.2	35.5	41.0	
**Trust in** **Institutions (3–12)**	7.52 (1.85)	7.58 (1.83)	7.37 (1.90)	t = 1.05
**Optimism (6–30)**	20.76 (4.49)	20.76 (4.45)	20.76 (4.58)	t = -0.01
**Mastery (7–35)**	25.92 (4.63)	26.11 (4.67)	25.51 (4.53)	t = 1.19
**Loneliness**				χ^2^ = 4.27
Infrequently-never	40.8	43.1	36.3	
Sometimes	38.6	39.2	37.1	
Often	20.6	17.7	26.6	
**Feeling more loneliness during COVID-19**				χ^2^ = 4.54 *
Yes	51.8	48.0	60.0	
No	48.2	52.0	40.0	

Note. * *p* < 0.05; ** *p* < 0.01.

**Table 2:** Psychological distress (PD) by the study variables (N = 389).

Variable	Psychological Distress Mean (SD)	*F*/t-test/Pearson Correlation
**Overall (7–28)**	15.57 (3.72)	
**Age**		F = 1.84
20–25	15.97 (4.22)	
26–30	15.49 (3.49)	
31–35	15.01 (3.14)	
**Gender**		t =2.02 *
Male	14.80 (4.09)	
Female	15.76 (3.61)	
**Marital status**		t = 3.97 **
Married	14.37 (2.99)	
Other	15.92 (3.85)	
**Have kids**		t = 2.51 *
Yes	14.71 (3.08)	
No	15.77 (3.83)	
**Education**		t = 0.86
Secondary education	15.78 (4.14)	
Post-secondary education	15.43 (3.44)	
**Parents’ education**		t = 2.48 *
Both academic	15.06 (3.61)	
Other	16.00 (3.77)	
**Currently studying**		t = −0.85
Yes	15.73 (3.86)	
No	15.41 (3.59)	
**Health problems/limitations**		t = −2.97 **
Yes	16.81 (3.71)	
No	15.32 (3.68)	
**Employment**		t = −3.82 **
Yes	15.08 (3.57)	
No	16.61 (3.84)	
**Perceived income adequacy in routine times**		t =−3.57 **
**Difficulties in covering monthly expenses**	17.39 (3.95)	
No difficulties in covering monthly expenses	15.32 (3.63)	
**Perceived income adequacy during COVID-19**		t = −5.71 **
Emerging difficulties in covering monthly expenses	16.89 (3.74)	
No difficulties in covering monthly expenses	14.75 (3.48)	
**Perceived trust**		t = 4.87 **
Yes	14.83 (3.41)	
No	16.73 (3.88)	
**Trust in** **institutions (3–12)**		r = −0.18 **
**Optimism (6–30)**		r = −0.52 **
**Mastery (7–35)**		r = −0.45 **
**Loneliness**		F = 107.11 **
Never-Infrequently	13.30 (2.83)	
Sometimes	16.01 (3.12) ^a^	
Often	19.25 (3.07) ^a,b^	
**Feeling more loneliness during COVID-19**		t = −6.99 **
Yes	16.86 (3.65)	
No	14.24 (3.45)	

Note. * *p* < 0.05; ** *p* < 0.01. ^a^ Significantly different (*p* < 0.001) from never-infrequently (Bonferroni post-hoc); ^b^ Significantly different (*p* < 0.001) from sometimes (Bonferroni post-hoc).

**Table 3:** Hierarchical multiple regression analysis for predicting PD (OLS).

Variable	Psychological Distress									
	Model 1		Model 2		Model 3		Model 4		Model 5	
	B (SE)	Β	B (SE)	β	B (SE)	β	B (SE)	β	B (SE)	β
**Age** (ref = 20–25)										
26–30	−0.10 (0.47)	−0.01	−0.11 (0.47)	−0.02	−0.21 (0.46)	−0.03	0.32 (0.39)	0.04	0.45 (0.35)	0.06
31–35	−0.03 (0.63)	0.00	0.10 (0.62)	0.01	0.15 (0.62)	0.02	0.44 (0.51)	0.05	0.43 (0.46)	0.05
**Gender** (male = 1)	−1.04 * (0.47)	−0.11	−0.95 * (0.46)	−0.10	−0.95 * (0.45)	−0.10	−1.45 *** (0.38)	−0.15	−1.16 ** (0.34)	−0.12
**Marital status** (married = 1)	−1.57 ** (0.71)	−0.18	−1.47 ** (0.70)	−0.16	−1.49 ** (0.69)	−0.17	−0.75 (0.57)	−0.08	−0.01 (0.52)	0.00
**Have kids** (yes = 1)	0.13 (0.74)	0.01	0.14 (0.73)	0.01	0.11 (0.72)	0.01	0.07 (0.60)	0.01	−0.28 (0.53)	−0.03
**Education** (post - secondary education = 1)	−0.05 (0.45)	−0.01	−0.04 (0.45)	−0.01	−0.09 (0.44)	−0.01	0.05 (0.37)	0.01	0.10 (0.33)	0.01
**Parents’ education** (both academic = 1)	−0.82 * (0.38)	−0.11	−0.79 * (0.37)	−0.11	−0.69 (0.37)	−0.09	−0.29 (0.31)	−0.04	−0.27 (0.28)	−0.04
**Health problems/ limitations** (yes = 1)	1.22 * (0.50)	0.12	1.23 * (0.49)	0.12	1.19 * (0.48)	0.12	0.24 (0.41)	0.02	0.18 (0.36)	0.02
**Perceived income adequacy in routine times** (difficulties in covering monthly expenses = 1)	1.56 ** (0.60)	0.14	1.52 * (0.59)	0.13	0.83 (0.62)	0.07	0.55 (0.51)	0.05	1.02 * (0.46)	0.09
**Unemployment** (=1)			1.31 ** (0.39)	0.16	0.70 (0.42)	0.09	0.87 * (0.35)	0.11	0.77 * (0.31)	0.10
**Perceived income adequacy during COVID-19** (difficulties in covering monthly expenses = 1)					1.50 ** (0.43)	0.20	0.90 * (0.36)	0.12	0.44 (0.33)	0.06
**Perceived trust**							−0.69 * (0.33)	−0.09	−0.78 ** (0.29)	−0.10
**Trust in institutions** (3–12)							−0.10 (0.08)	-0.05	0.09 (0.07)	−0.05
**Mastery** (7–35)							−0.17 *** (0.04)	−0.21	−0.12 ** (0.03)	−0.14
**Optimism** (6–30)							−0.32 *** (0.04)	−0.39	−0.22 *** (0.04)	−0.27
**Loneliness** (ref = never-infrequently)										
Sometimes									1.40 *** (0.35)	0.18
Often									3.41 *** (0.46)	0.37
**Feeling more loneliness during COVID-19** (yes = 1)									0.78 * (0.32)	0.10
**Constant**	16.17 *** (0.42)		15.68 *** (0.44)		15.40 *** (0.44)		27.38 *** (1.14)		22.33 *** (1.16)	
**R^2^**	0.098		0.124		0.152		0.425		0.548	
**R** ^**2**^ **Change**	0.098 ***		0.026 **		0.028 **		0.274 **		0.123 ***	

Note. * *p* < 0.05; ** *p* < 0.01; *** *p* < 0.001.; SE = standard error; OLS = ordinary least squares, ref = reference group.
